# Distinct structural motifs are necessary for targeting and import of Tim17 in *Trypanosoma brucei* mitochondrion

**DOI:** 10.1128/msphere.00558-23

**Published:** 2024-01-09

**Authors:** Chauncey Darden, Joseph E. Donkor, Olga Korolkova, Muhammad Younas Khan Barozai, Minu Chaudhuri

**Affiliations:** 1 Department of Biochemistry, Cancer Biology, Neuroscience, and Pharmacology, Meharry Medical College, Nashville, Tennessee, USA; 2 Department of Microbiology, Immunology, and Physiology, Meharry Medical College, Nashville, Tennessee, USA; 3 The Consolidated Research Instrumentation, Informatics, Statistics, and Learning Integration Suite (CRISALIS), Meharry Medical College, Nashville, Tennessee, USA; 4 Center for AIDS Health Disparities Research, Meharry Medical College, Nashville, Tennessee, USA; Cleveland State University, Cleveland, Ohio, USA

**Keywords:** mitochondria, TbTim17, *Trypanosoma brucei*, targeting signal, protein import

## Abstract

Nuclear-encoded mitochondrial proteins are correctly translocated to their proper sub-mitochondrial destination using location-specific mitochondrial targeting signals and via multi-protein import machineries (translocases) in the outer and inner mitochondrial membranes (TOM and TIMs, respectively). However, targeting signals of multi-pass Tims are less defined. Here, we report the characterization of the targeting signals of *Trypanosoma brucei* Tim17 (TbTim17), an essential component of the most divergent TIM complex. TbTim17 possesses a characteristic secondary structure including four predicted transmembrane (TM) domains in the center with hydrophilic N- and C-termini. After examining mitochondrial localization of various deletion and site-directed mutants of TbTim17 in *T. brucei* using subcellular fractionation and confocal microscopy, we located at least two internal targeting signals (ITS): (i) within TM1 (31–50 AAs) and (ii) TM4 + loop 3 (120–136 AAs). Both signals are required for proper targeting and integration of TbTim17 in the membrane. Furthermore, a positively charged residue (K^122^) is critical for mitochondrial localization of TbTim17. This is the first report of characterizing the ITS for a multipass inner membrane protein in a divergent eukaryote, like *T. brucei*.

**IMPORTANCE:**

African trypanosomiasis (AT) is a deadly disease in human and domestic animals, caused by the parasitic protozoan *Trypanosoma brucei*. Therefore, AT is not only a concern for human health but also for economic development in the vast area of sub-Saharan Africa. *T. brucei* possesses a single mitochondrion per cell that imports hundreds of nuclear-encoded mitochondrial proteins for its functions. *T. brucei* Tim17 (TbTim17), an essential component of the TbTIM17 complex, is a nuclear-encoded protein; thus, it is necessary to be imported from the cytosol to form the TbTIM17 complex. Here, we demonstrated that the internal targeting signals within the transmembrane 1 (TM1) and TM4 with loop 3, and residue K122 are required collectively for import and integration of TbTim17 in the *T. brucei* mitochondrion. This information could be utilized to block TbTim17 function and parasite growth.

## INTRODUCTION

Mitochondria are essential organelles in eukaryotes. This is not only because mitochondria produce 80% of cellular ATP but also for their roles in various cellular functions, e.g., calcium homeostasis, programmed cell death, antiviral immunity, and cell signaling (1–4). Mitochondrial defects cause many human diseases, particularly several devastating neurodegenerative disorders including Alzheimer’s disease, Parkinson’s disease, and others (5–9). In order for mitochondria to perform such diverse functions, close to 1,000 nuclear-encoded proteins have to be imported into the mitochondria from the cytosol (10, 11). Mitochondria possess an intricate machinery to import these nuclear-encoded proteins and to sort them into proper sub-mitochondrial destinations, such as mitochondrial outer and inner membranes (MOM and MIM, respectively), intermembrane space (IMS), and matrix (12, 13).

Mitochondrial import machinery has long been studied in yeast, humans, and plants (14–16). In these organisms, the MOM possesses one translocase, the TOM complex that imports almost all nuclear-encoded mitochondrial proteins (17). Whereas, the mitochondrial inner membrane MIM has two major translocases, TIM23 and TIM22 (12, 13). Likewise, TOM and TIMs are multi-subunit protein complexes. Mitochondrial matrix proteins with N-terminal targeting signals (MTS), also referred to as the presequence, are translocated from the TOM to the TIM23 complex (18, 19). Whereas, polytopic mitochondrial inner membrane proteins are translocated via the TIM22 complex (20, 21). Most of these proteins do not have a MTS, they use internal targeting signals (ITS) for their import into mitochondria. These proteins are chaperoned from the TOM to the TIM22 complex via small Tim complexes in the IMS (22, 23). The Tim23 and Tim22 proteins are known as the channel-forming unit of the TIM23 and TIM22 complexes, respectively (24, 25). Tim17 interacts with Tim23, and it was thought that it acts as a structural component of the TIM23 translocase and regulates its activity (26). However, recent studies proposed that Tim17 is the major subunit of the presequence translocase and is involved in the translocation of the preprotein across the MIM (27). Mitochondrial membrane potential is required for protein translocation through either the TIM23 or TIM22 complexes (12, 13). In spite of elaborate discoveries of mitochondrial protein import machinery in fungi and mammals, protein complexes involved in similar functions in early divergent eukaryotes have just begun to emerge (28, 29).

*Trypanosoma brucei* is a parasitic protozoan that belongs to a group of very early divergent eukaryotes. *T. brucei* causes a fatal disease in humans known as African sleeping sickness and a similar disease in livestock, called Nagana (30, 31). One of the several unique characteristics of *T. brucei* is that it possesses a single mitochondrion per cell that is elongated along the cell body (32, 33). Mitochondrial DNA in this organism is a concatenated structure of many circular DNAs known as kinetoplast (34). In spite of the complex structure of this mitochondrial genome, it encodes only 18 proteins, the rest of the mitochondrial proteins, which are about ~900, are nuclear encoded and therefore imported into the mitochondrion similar to other eukaryotes (35). However, it has been shown that mitochondrial protein import machinery in *T. brucei* is quite divergent (28, 29, 36, 37). The ATOM complex in the MOM of *T. brucei* consists of several trypanosome-specific proteins along with Atom40, the archaic homolog of Tom40 (38). It also performs similar functions of the TOM complex. *T. brucei* MIM possesses a single TIM complex that presumably imports presequence-containing matrix proteins as well as the polytopic MIM proteins (37, 39). The major component of the *T. brucei* TIM (TbTIM) complex is TbTim17, which is the homolog of the Tim17, Tim22, and Tim23 family of proteins in fungi and mammals. Additionally, TbTim17 is the single homolog of this family found in *T. brucei* (36, 40). It associates with several unique Tim proteins to form a modular protein complex, which on a native gel separates into multiple complexes within the range of 300 to 1,100 kDa (36, 40). The predicted secondary structure of TbTim17 is very similar to fungal and mammalian Tim17/Tim22/Tim23 proteins (40). TbTim17 has four predicted transmembrane (TM) domains, and the N- and C-terminal regions are hydrophilic, exposed in the IMS. Although the length of these regions and the loops between two consecutive TMs vary among different homologs in different species. Furthermore, the primary sequence of TbTim17 only shows 20% to 30% similarity to its homologs in other systems, which in contrast 50% to 60% homology among the yeast and mammalian Tim17/22/23 proteins (41). Therefore, how this single homolog in *T. brucei* performs the functions that is done by three isomeric proteins is the subject of intense study.

Similar to other nuclear-encoded mitochondrial proteins, TbTim17 is imported into the *T. brucei* mitochondrion and assembled in the TbTIM complex. As a polytopic MIM protein, TbTim17 does not have any predicted N-terminal targeting signal. It also has been shown that the deletion of the first 30 amino acid residues (AAs) did not hamper the import of TbTim17 into *T. brucei* mitochondrion (42). Therefore, TbTim17 must depend on internal targeting signal(s) for its import. Multiple studies were performed to identify the ITS of *Saccharomyces cerevisiae* (Sc) Tim17 and ScTim23 (43, 44). Results indicated that the region within the C-terminal half of these proteins, 181–222 AAs of ScTim23 and 102–158 AAs of ScTim17, is required for import and insertion into the MIM (43). Furthermore, these C-terminal regions possess a stretch of amino acid sequence containing few positively charged and hydroxylated residues, and this sequence was capable to import a reporter protein into the matrix (43). From these studies, authors speculated that this region, which is in between the third and fourth TMs, is the import signal for ScTim17 and ScTim23. However, later studies revealed that the charged residues either in loop 1 or loop 3 of ScTim23 are not required for its import into mitochondria but needed for insertion of this protein into the MIM. Alternatively, a pair of TMs, particularly the first and fourth, is required for efficient import of ScTim23 (44).

Since *T. brucei* possesses a divergent import machinery and TbTim17 is assembled with non-canonical TbTims, it is necessary to investigate the import process of this essential translocator protein in this parasite. Here, we systematically created a series of truncation and several point mutations of TbTim17 to analyze the structural motifs needed for import and insertion of this protein into *T. brucei* MIM. Our results showed that in spite of some similarities with the previous findings found in yeast Tim17/Tim23, a few distinct features of TbTim17 are critical for import of this essential protein into the *T. brucei* mitochondrion. These results also indicate a possible evolutionary difference in the import process of the multi-pass MIM proteins. Import signals have not been investigated for any of the polytopic MIM proteins in kinetoplastid organisms; therefore, this knowledge can be applicable for such proteins in *T. brucei* as well as in other kinetoplastid parasites that also cause devastating diseases in humans.

## RESULTS

### N- and C-terminal deletion mutations revealed the importance of TM1 and TM4 for import of TbTim17 into mitochondria

**Fig 1:**
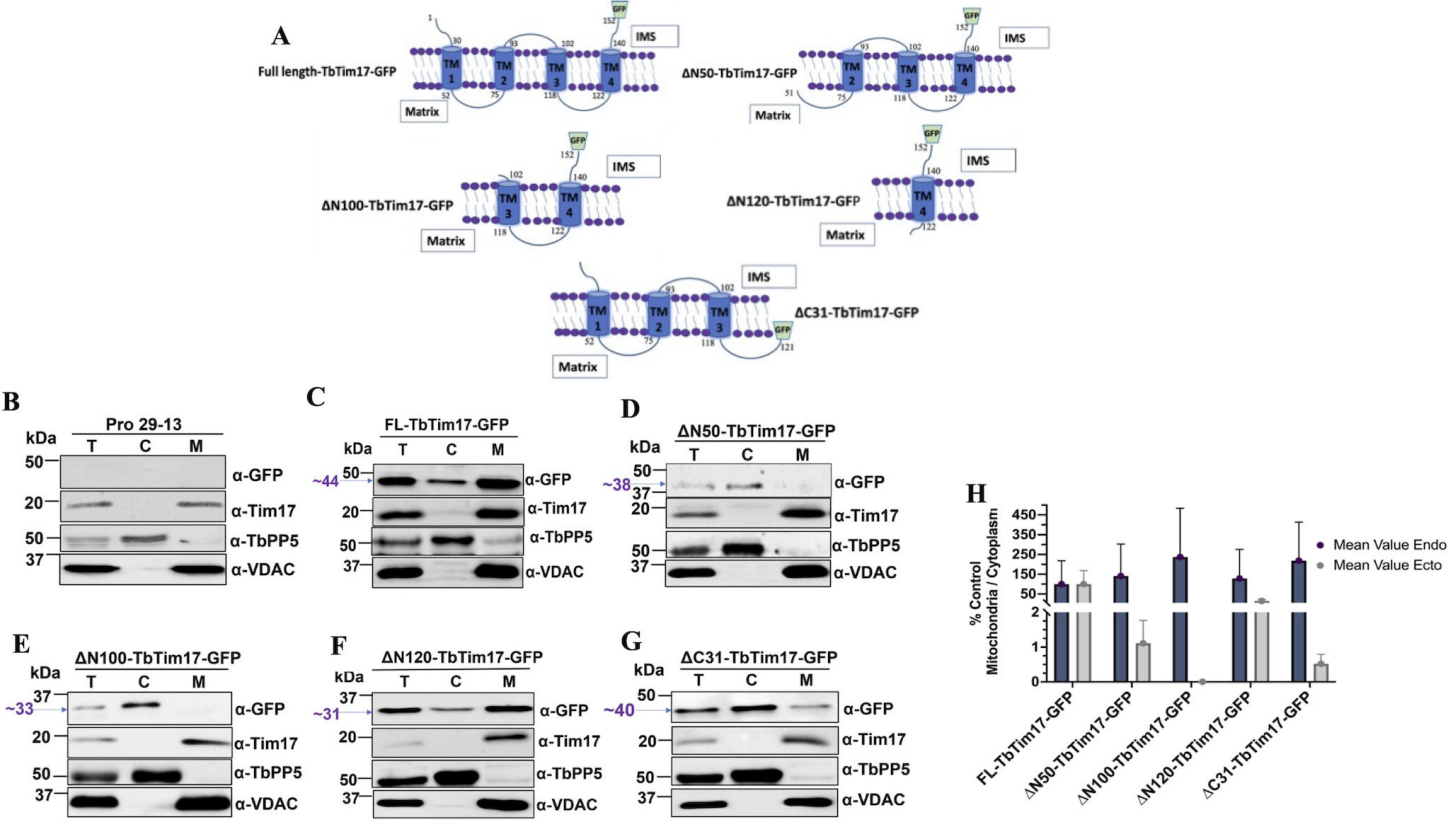
Effect of the N- and C-terminal deletion mutations on sub-cellular localization of TbTim17. (**A**) Schematic of N- and C-terminal deletion mutants. The full-length and mutant proteins are drawn according to the predicted membrane topology. The transmembrane domains are represented by blue cylinders and labeled 1–4. Numbers indicate the position of the amino acid residues. GFP tag is shown by a green trapezoid. (**B–G**) Immunoblot analysis of the sub-cellular fractions of the *T. brucei* expressing FL and mutant TbTim17. The parental cell line (29-13) was used in parallel as the control (**B**). The stable transfectants of *T. brucei* with the FL and mutant constructs, FL-TbTim17-GFP (**C**), ∆N50-TbTim17-GFP (**D**), ∆N100-TbTim17-GFP (**E**), ∆N120-TbTim17-GFP (**F**), and ∆C31-TbTim17-GFP (**G**) were induced for ~40–45 h, and sub-cellular fractions were collected. Proteins in the total (T), cytosolic (C), and mitochondrial (M) fractions were analyzed by immunoblot using antibodies for GFP, TbTim17, TbPP5, and VDAC. We found similar results for a shorter induction period (18–20 h). (**H**) Densitometric analysis of the immunoblot results. Intensity of the GFP-fusion protein bands in the mitochondrial and cytosolic fractions was measured by Image Lab software (Bio Rad), normalized with the intensities for FL-TbTim17-GFP in the respective fractions. Densitometric analysis of the endogenous (TbTim17) in the cytosolic and mitochondrial fractions was performed in parallel. The ratio of the mitochondrial vs cytosolic fractions for both endogenous (dark blue circle) and ectopic (gray circle) was calculated as the percent of the full-length protein and plotted against different cell types using GraphPad Prism. Error bars represent SEM for each data group. Sample size: *n* = 3 (in average).

It has been shown previously that the first 30 AAs, the N-terminal hydrophilic region, of TbTim17 are not required for import of this protein into the mitochondrion in *T. brucei* (42). To determine the ITS, we created a series of additional deletion mutants of TbTim17 that removed TM1 (ΔN50), TM1-TM2 (ΔN100), TM1-TM3 (ΔN120), and TM4 (ΔC31). Each mutant protein was attached to green fluorescence protein (GFP) at the C-terminal end for localization tracking in the cell (Fig. 1A). The mutant proteins were expressed in *T. brucei* from a tetracycline-inducible expression vector. The full-length (FL) TbTim17 was also attached with GFP at the C-terminal end (Fig. 1A) and expressed in *T. brucei* in a similar manner. After induction of expression, cells were harvested, and sub-cellular fractions were isolated. Proteins in the total, cytosolic, and mitochondrial fractions were analyzed by TbTim17 and GFP antibodies. The cytosolic and mitochondrial marker proteins, *T. brucei* protein phosphatase 5 (TbPP5) and voltage-dependent anion channel (VDAC), respectively, were detected by specific antibodies, as controls. The parental *T. brucei* cell lines (Pro 29–13) were used in parallel (Fig. 1B). Results showed that the FL-TbTim17-GFP was mostly present in the mitochondrial fraction (Fig. 1C). A smaller portion was found in the cytosolic fraction, which is likely due to overexpression of the protein. We attempted to express the N-terminal GFP-tagged TbTim17 but failed to see any expressed protein, indicating that the N-terminal GFP-tagged TbTim17 is likely unstable. It has been shown previously that the C-terminally tagged TbTim17 (TbTim17-2X-Myc and TbTim17-tandem affinity purification tag) were properly targeted to *T. brucei* mitochondria (36). Therefore, we do not think tagging at the C-terminal with GFP has any adverse effect on import. In contrast to the FL-TbTim17, ΔN50-TbTim17-GFP and ΔN100-TbTim17-GFP were not present in the mitochondrial fraction but found in the cytosolic fraction (Fig. 1D and E), indicating that deletion of TM1 and TM1-TM2 hampered import of TbTim17 into mitochondria in *T. brucei*. In comparison to the FL-TbTim17-GFP, the mutant proteins were expressed less, which is likely due to the degradation of the unimported proteins in the cytosol. Interestingly, ΔN120-TbTim17-GFP was primarily localized in mitochondria, showing that the C-terminal region (121–152 AAs), including loop 3, TM4, and the C-terminal hydrophilic region, is capable to localize GFP into mitochondria (Fig. 1F). Furthermore, when we deleted this region, the mutant protein, ΔC31 (1–120 AAs)-TbTim17-GFP, stayed primarily in the cytosol (Fig. 1G). We did not observe any differences in the localization of the endogenous TbTim17 in the mutant cell lines (Fig. 1B through G). Similarly, TbPP5 and VDAC were found primarily in the cytosolic and mitochondrial fractions, respectively, in all cell lines, as expected. We quantitated the ratio of mitochondrial/cytosolic localization of each mutant relative to the FL-TbTim17. Results from three independent experiments revealed that deletion of the first 50 or 100 AAs of TbTim17 reduced mitochondrial localization of these mutants >98% and 100%, respectively, in comparison to the FL-TbTim17-GFP (Fig. 1H). In contrast, ΔN120-TbTim17-GFP localized to the mitochondria ~50% in comparison to the FL-TbTim17-GFP (Fig. 1H). In addition, deletion of this C-terminal region, ΔC31-TbTim17-GFP, hampered its localization into mitochondria >99%. These results strongly suggest that the C-terminal 31 AAs must have some targeting information. However, it was puzzling to see that this region is present in the ΔN50-TbTim17-GFP and ΔN100-TbTim17-GFP mutants, but these mutants were not imported into mitochondria, which indicates two possibilities: (i) besides the C-terminal 31 AAs, additional regions in the first two TMs are necessary for importing TbTim17, or (ii) truncation of TM1 and TM1-TM2 changed the conformation of the protein, so the C-terminal importing signal was not exposed. To understand, if the truncation mutations have an effect on the overall structure of the protein, we have performed *in silico* structural modeling using (i) RaptorX program (https://bio.tools>raptorx) with no template and (ii) Swiss modeling (https://swissmodel.expasy.org/) based on the cryo-EM structure of the human Tim22 (Fig. S1A and B). From both analyses, we found that the remaining overall structure of TbTim17 after truncation of TM1 and TM2 is somewhat maintained. Furthermore, similar truncation mutants of ScTim23 have been used for import studies (44). Together, our results indicate the presence of more than one targeting signal in TbTim17. As we found deletion of the first 30 AAs did not, but deletion of the first 50 AAs, hampered the import of TbTim17 significantly, we estimated that TM1 (31–50 AAs) likely harbors a targeting signal. Therefore, these initial results suggest the presence of possible ITSs within at least TM1 and the C-terminal region, including TM4.

**Fig 2:**
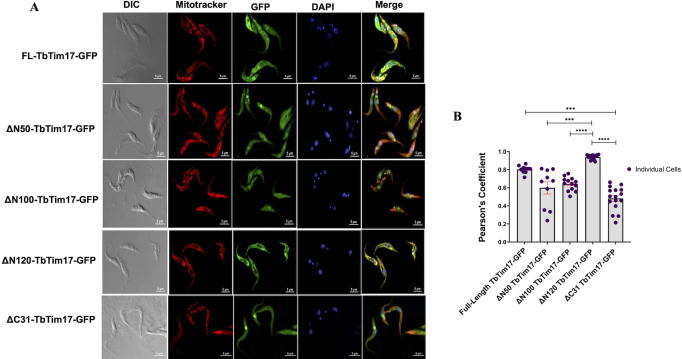
Immunofluorescence microscopy of *T. brucei*-expressed FL and mutant TbTim17. (**A**) The FL-TbTim17-GFP and the mutant cell lines (∆N50-, ∆N100-, ∆N120-, and ∆C31-TbTim17-GFP) were induced with doxycycline for 18–20 h. Cells were harvested and stained with MitoTracker Red to visualize the mitochondrion. Expression of GFP fusion proteins is seen by green fluorescence, DAPI was used to stain the nucleus and kinetoplast (mitochondrial genome), and phase-contrast pictures (DIC) are shown. Merge images show colocalization. (**B**) Pearson’s coefficient values were calculated from the merge images and plotted for each type of cells using GraphPad Prism. Individual data points, *n* = 10 (FL-Tim17 and ∆N50), *n* = 13 (∆N100), and *n* = 16 (∆N120 and ∆C31), are shown. Error bars represent SEM for each data group. Kruskal-Wallis statistical test was performed on all data sets; *** indicates *P* = 0.0003 (∆N50 vs ∆N120), *P* = 0.0008 (FL-17 vs ∆C31); **** indicates *P* < 0.0001.

To confirm our immunoblot data, we tracked the localization patterns of TbTim17 deletion mutants tagged with GFP at the C-terminal end in *T. brucei* by confocal microscopy (Fig. 2A). Mitochondria were stained with MitoTracker Red, which is a fluorescent dye that incorporates into mitochondria in a membrane potential-dependent manner (45). We induced the FL and mutant TbTim17-GFP cell lines for ~18–20 h before staining with MitoTracker Red. We used the FL-TbTim17-GFP cell line to optimize the induction period to have sufficient expression for localization of this protein, then used the same time point for all other mutants. 4′,6-Diamidino-2-phenylindole (DAPI) was used to stain the nuclear and mitochondrial DNAs in *T. brucei*. Afterward, merged pictures were used to visualize colocalization of the expressed GFP-tagged proteins with MitoTracker Red in mitochondria. We have also calculated the Pearson’s coefficient (PC) values of 15–20 individual cells for quantifying the extent of colocalization (Fig. 2B). Our results showed that the FL-Tim17-GFP protein colocalized well with the MitoTracker Red-stained mitochondria, in most of the cells as expected, with an average PC value of 0.8 (Fig. 2A and B). In contrast, we did not observe colocalization of ΔN50-TbTim17-GFP, ΔN100-TbTim17-GFP, and ΔC31-TbTim17-GFP with MitoTracker Red as most of the GFP-labeled protein was found in the cytosol of the cell (Fig. 2A). Average PC values for these three mutants were 0.58, 0.65, and 0.5, respectively (Fig. 2B). To this end, PC values near 1.0 are considered as colocalization (46). Therefore, PC values near 0.5 or below are considered as least colocalization. These results were correlated with our western blot data shown in Fig. 1B. On the other hand, ΔN120-TbTim17-GFP was colocalized with MitoTracker Red even better than the FL-TbTim17-GFP (Fig. 2A). The average PC value for ΔN120-TbTim17-GFP was 0.95 (Fig. 2B). We noticed that ΔN50-TbTim17-GFP, ΔN100-TbTim17-GFP, and ΔC31-(1–120 AAs)-TbTim17-GFP mutant proteins were accumulated in some kind of vesicles located in the cytosol of some cells (Fig. 2A). In many cases, these vesicles were adjacent to the nucleus; however, it requires further investigation to confirm this aberrant location of the mutant proteins. Together, our results further confirmed the importance of TM1 and TM4 of TbTim17 for its localization into mitochondria in *T. brucei*. Particularly, we found that the C-terminal region (121–152 AAs) of TbTim17 was capable of importing GFP into the mitochondria in *T. brucei*, suggesting the presence of an ITS in this region.

### The ΔN120-TbTim17-GFP was located inside the mitochondria

**Fig 3:**
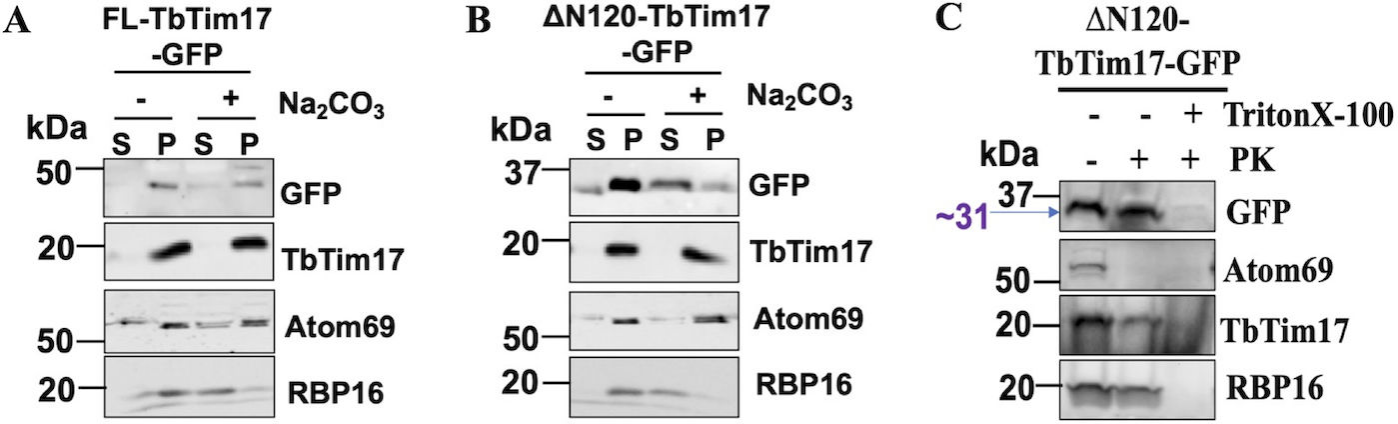
Sub-mitochondrial location of ∆N120-TbTim17-GFP. (A and B) Alkali extraction of mitochondria isolated from *T.brucei*-expressed FL-TbTim17-GFP (**A**) and ∆N120-TbTim17-GFP (**B**). Mitochondria were treated (+) and left untreated (−) with Na_2_CO_3_, pH 11.0. The soluble (S) and insoluble pellet (P) fractions were separated by centrifugation and analyzed by immunoblot analysis using antibodies as indicated. (**C**) Limited protease digestion of mitochondria isolated from *T. brucei*-expressed ∆N120-TbTim17-GFP. Mitochondria were treated (+) or left untreated (−) with proteinase K (PK) and TritonX-100 as shown. After treatment, mitochondria were recovered by centrifugation, and proteins were analyzed by immunoblot using antibodies as indicated. Each experiment was repeated three times, and representative blots are shown.

Next, we wanted to see if ΔN120-TbTim17-GFP is integrated into the mitochondrial membrane like the endogenous TbTim17. To investigate this, we isolated mitochondria from both FL-Tim17-GFP and ΔN120-TbTim17-GFP cell lines, treated them with 0.1 M sodium carbonate at pH 11.5, and separated the soluble and pellet fractions by centrifugation. Extraction with sodium carbonate solubilizes peripherally associated membrane proteins and matrix and IMS proteins, but membrane integral proteins stay in the membrane pellet (47). After centrifugation, the supernatant and the pellet fractions obtained from both the ΔN120-TbTim17-GFP and FL-Tim17-GFP mitochondrion samples were analyzed by SDS-PAGE and immunoblot analysis using GFP and Tim17 antibodies to detect both ectopic and endogenous TbTim17 proteins, respectively (Fig. 3A and B). We also used antibodies for Atom69, which is a membrane-anchored component of the ATOM complex in the outer membrane, and RNA-binding protein 16 (RBP16), which is a matrix protein. As expected, upon treatment with sodium carbonate, endogenous TbTim17 protein in both cell lines was found only in the pellet fraction, indicating full integration into the mitochondrial membrane (Fig. 3A and B). Likewise, FL-Tim17-GFP protein was also found mostly in the pellet fraction, although a tiny amount was released into the supernatant fraction (Fig. 3A). On the other hand, a larger portion of ΔN120-TbTim17-GFP was found in the supernatant, and only a tiny portion was located within the membranous pellet fraction (Fig. 3B), suggesting ΔN120-TbTim17-GFP was a soluble protein and did not integrate into the mitochondrial membrane. Therefore, although targeting GFP to mitochondria, the C-terminal hydrophilic region in conjunction with TM4 was not able to integrate the fusion protein into the membrane. It is likely that ΔN120-TbTim17-GFP is either peripherally attached to MOM and not imported into the mitochondria, or it is being mis-targeted to the IMS or matrix. As expected, Atom69 was found in the pellet fractions in both cell lines as an integral membrane protein (Fig. 3A and B). Similarly, as expected, RBP16 was found primarily in the soluble fraction (Fig. 3A and B).

To investigate if ΔN120-TbTim17-GFP is peripherally associated with the cytosolic face of the MOM or it enters into the mitochondria, we treated isolated mitochondria with proteinase K (PK; 100 µg/µL). After treatment, mitochondria were pelleted by centrifugation, and proteins were analyzed by western blotting using antibodies for GFP, TbTim17, Atom69, and RBP-16 (Fig. 3C). We also treated a separate portion of mitochondria with proteinase K (100 µg/µL) in the presence of Triton X-100 (1%), a non-ionic detergent, and analyzed in a similar manner. ΔN120-TbTim17-GFP was protected from digestion of proteinase K when the mitochondrial membrane was kept intact, which is similar to endogenous TbTim17 and RBP16, (Fig. 3C). In the presence of Triton X-100, proteins were digested by proteinase K, showing that the protected bands were not generated due to protease resistance. Whereas, Atom69 was completely digested by proteinase K, as expected (Fig. 3C). Together, these results showed that ΔN120-TbTim17-GFP was imported into the mitochondria but stays as a soluble IMS or matrix protein.

It has been reported previously that ScTim17 and ScTim23, each possesses a presequence-like sequence within the TM3 and TM4 (43). Therefore, we investigated if TbTim17 also possesses a similar signal. To determine this, we used TargetP 2.0, a software prediction program that was designed to identify subcellular localizations of proteins based on the presence of N-terminal presequences (mitochondrial, chloroplast, and secretory/ER-targeting signals) found in the primary amino acid sequence (48). We analyzed residues 120–152 AAs of TbTim17 using this program and found that it has ~41% probability for being a mitochondrial transfer peptide, ~48% probability to act as an ER signal peptide, and ~11% were listed as other (Fig. S2). The primary sequence of this region consists of multiple hydrophobic residues and a few charged residues (Fig. S2). Therefore, it is possible that this region being in front of GFP acted as a presequence-like targeting signal and translocated GFP into the matrix. Although ΔN120-TbTim17-GFP possesses TM4 of TbTim17, this TM was unable to anchor the protein in the membrane.

### TM1 alone could target GFP into mitochondria, but the protein was not properly integrated into the mitochondrial membrane

**Fig 4:**
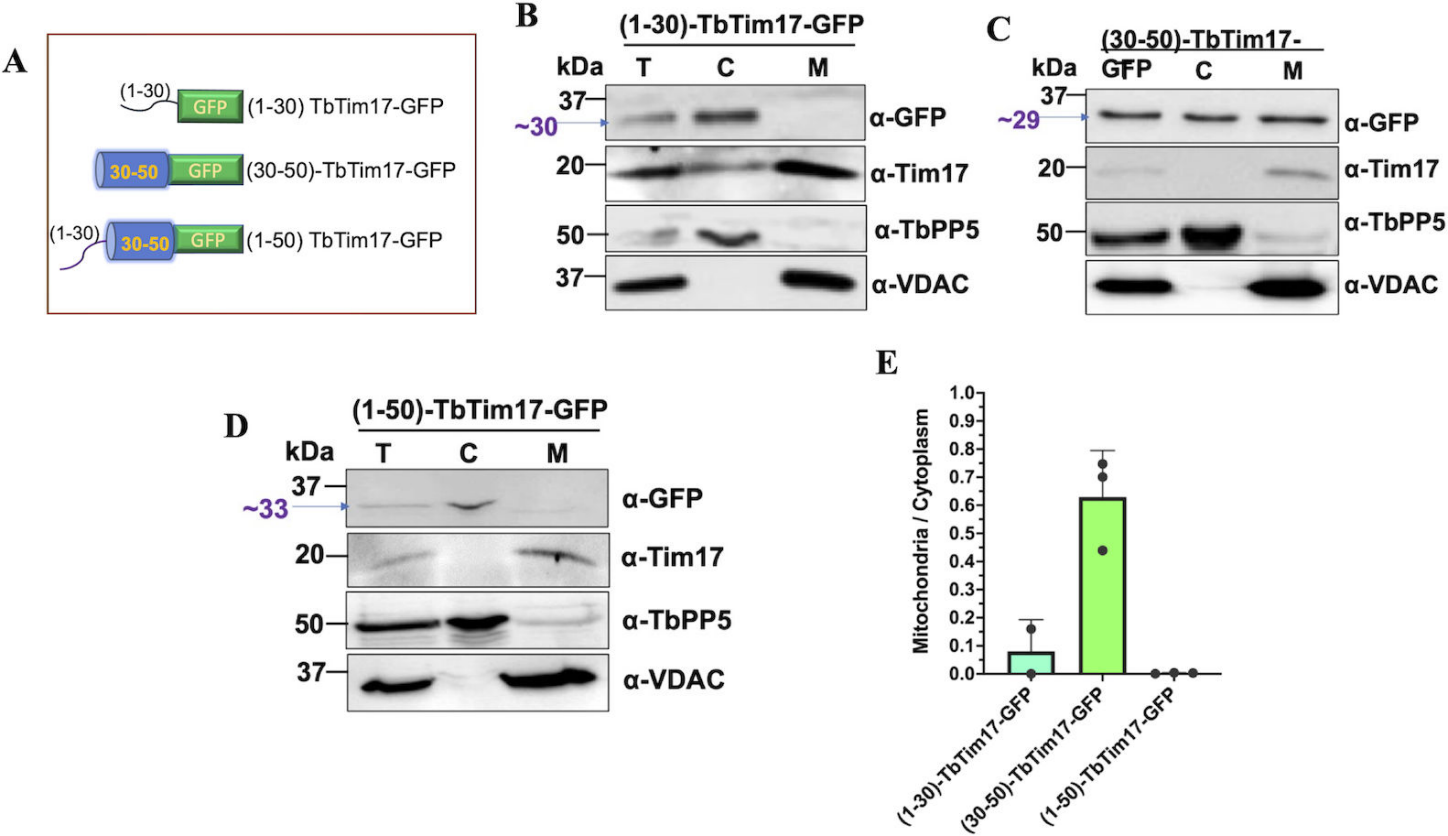
Analysis of the sub-cellular locations of the reporter protein containing TbTim17 N-terminal hydrophilic region, TM1, and both. (**A**) Schematics of the 1–30-, 30–50- and 1–50-TbTim17 tagged with GFP constructs. The N-terminal helical region, TM1, and GFP are shown in black line, blue cylinder, and green rectangle, respectively. (**B**) Immunoblot analysis of the subcellular fractions, total (T), cytosol (C), and mitochondria (M), of *T. brucei* expressing the (1–30)-TbTim17-GFP (**B**), (30–50)-TbTim17-GFP (**C**), and (1–50)-TbTim17-GFP (**D**) fusion proteins using antibodies for GFP, TbTim17, TbPP5, and VDAC. (**E**) Densitometric analysis of the immunoblot results. Intensity of the GFP-fusion protein bands in the mitochondrial and cytosolic fractions was measured by Image Lab software (Bio Rad). The ratio of the mitochondrial vs cytosolic fractions was calculated and plotted against different cell types using GraphPad Prism. Error bars represent SEM for each data group. Sample size: *n* = 3 (in average).

As we observed that the removal of the first 30 AAs’ hydrophilic region of TbTim17 did not hamper its localization to mitochondria, but deletion of the first 50 AAs, which include TM1, totally inhibits the import of TbTim17 into the mitochondria (Fig. 1 and 2), we decided to investigate if the region, 30–50 AAs, alone could target GFP into the mitochondria. For this purpose, we created three additional protein constructs, such as (i) 1–30 AAs, the N-terminal hydrophilic region of TbTim17, fused with GFP [(1–30)-TbTim17-GFP], (ii) 30–50 AAs, which comprise TM1 only, linked with GFP [(30–50)-TbTim17-GFP], and (iii) 1–50 AAs of TbTim17 fused with GFP [(1–50)-TbTim17-GFP] (Fig. 4A). These fusion proteins were expressed from an inducible expression vector as described for other mutants, and sub-cellular location of these proteins was assessed by immunoblot analysis and confocal microscopy. Immunoblot analysis of the sub-cellular fractions showed that (1–30)-TbTim17-GFP was present in the cytosolic fraction (Fig. 4B), further confirming that TbTim17 does not contain an N-terminal localization signal. Conversely, the (30–50)-TbTim17-GFP fusion protein was localized to the mitochondria at least partially (Fig. 4C). Surprisingly, 1–50 AAs of TbTim17 failed to localize GFP to the mitochondria despite containing TM1 (Fig. 4D). We quantitated the ratio of mitochondrial/cytosolic localization of each mutant, and results showed that about 60% of the (30–50)-TbTim17-GFP was enriched in the mitochondrial fraction, whereas enrichment of (1–30)-TbTim17-GFP and (1–50)-TbTim17-GFP was less than 5% (Fig. 4E). Together, these results indicated that TM1, indeed, contains an ITS, whereas (1–50)-TbTim17-GFP mutant could be folded differently, thus the signal was poorly recognized. Using TargetP software program, we analyzed the sequence of the (1–30), (30–50), and (1–50) regions of TbTim17 (Fig. S3). Interestingly, we found that the (30–50) region of TbTim17 has a higher probability (84%) of being a signal peptide, and peak was observed at the region of 45–50 AAs; however, when we analyzed (1–50) region of TbTim17, we found a lower probability score (15%), and we did not see any probability peak, suggesting that the (30–50) region must be at the N-terminus of the protein to act as a targeting signal. These predictions were well correlated with our experimental results regarding mitochondrial targeting of these fusion constructs.

**Fig 5:**
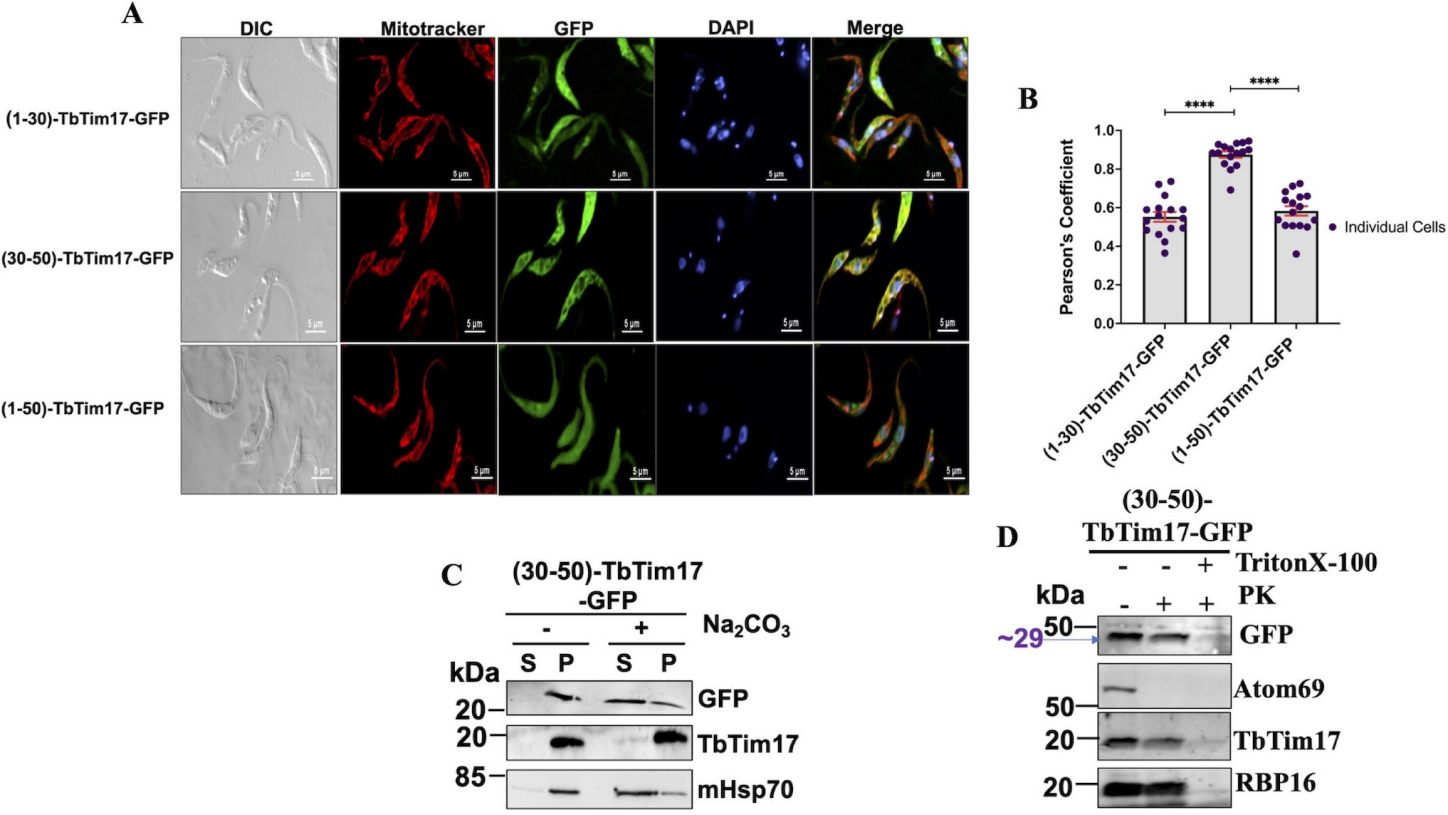
Immunofluorescence microscopy images of *T. brucei*-expressed reporter protein containing TbTim17 N-terminal hydrophilic region, TM1, and both. (**A**) *T. brucei* cell lines, (1–30)-TbTim17-GFP, (30–50)-TbTim17-GFP, and (1–50)-TbTim17-GFP, were induced with doxycycline for 18–20 h. Cells were harvested and stained with MitoTracker Red to visualize the mitochondrion. Expression of GFP fusion proteins was seen by green fluorescence, DAPI was used to stain the nucleus and kinetoplast (mitochondrial genome), and phase-contrast pictures (DIC) are shown. Merge images show colocalization. (**B**) Pearson’s coefficient values were calculated from the merge images and plotted for each type of cells using GraphPad Prism. Individual data points (*n* = 10–20) are shown. Error bars represent SEM for each data group. Statistical significance was calculated by Kruskal-Wallis statistical test; **** indicates *P* < 0.0001. (**C**) Alkali extraction of mitochondria isolated from *T.brucei*-expressed (30–50)-TbTim17-GFP. Mitochondria were treated (+) and left untreated (−) with Na_2_CO_3_, pH 11.0. The soluble (S) and insoluble pellet (P) fractions were separated by centrifugation and analyzed by immunoblot analysis using antibodies as indicated. (**D**) Limited protease digestion of mitochondria isolated from *T. brucei*-expressed (30–50)-TbTim17-GFP. Mitochondria were treated (+) or left untreated (−) with proteinase K and TritonX-100 as shown. After treatment, mitochondria were recovered by centrifugation, and proteins were analyzed by immunoblot using antibodies as indicated. Each experiment was repeated three times, and representative blots are shown.

We verified our western blot data with confocal microscopy (Fig. 5A). We found that the (30–50)-TbTim17-GFP colocalized with Mitotracker with an average PC value around 0.82 (Fig. 5A and B). Whereas, (1–30)-TbTim17-GFP and (1–50)-TbTim17-GFP were mostly localized in the cytosol (Fig. 5A and B), with an average PC value of 0.5 (Fig. 5B). Next, we examined if (30–50)-TbTim17-GFP was integrated into the mitochondrial membrane. Alkali extraction followed by immunoblot analysis of the soluble and pellet fractions showed that a majority of the (30–50)-TbTim17-GFP was present in the supernatant and a smaller fraction stays in the pellet, indicating that the protein was partially integrated into the mitochondrial membrane (Fig. 5C). Therefore, TM1 alone was not able to anchor strongly the fusion protein in the membrane. As described above, we performed PK protection assay to assess the sub-mitochondrial location of the (30–50)-TbTim17-GFP. We found that this mutant protein is well protected from PK digestion (Fig. 5D), indicating that it crossed the MOM.

### Deletion of the C-terminal hydrophilic regions and a part of TM4 did not hamper targeting but inhibits the import and integration of TbTim17 into the MIM

**Fig 6:**
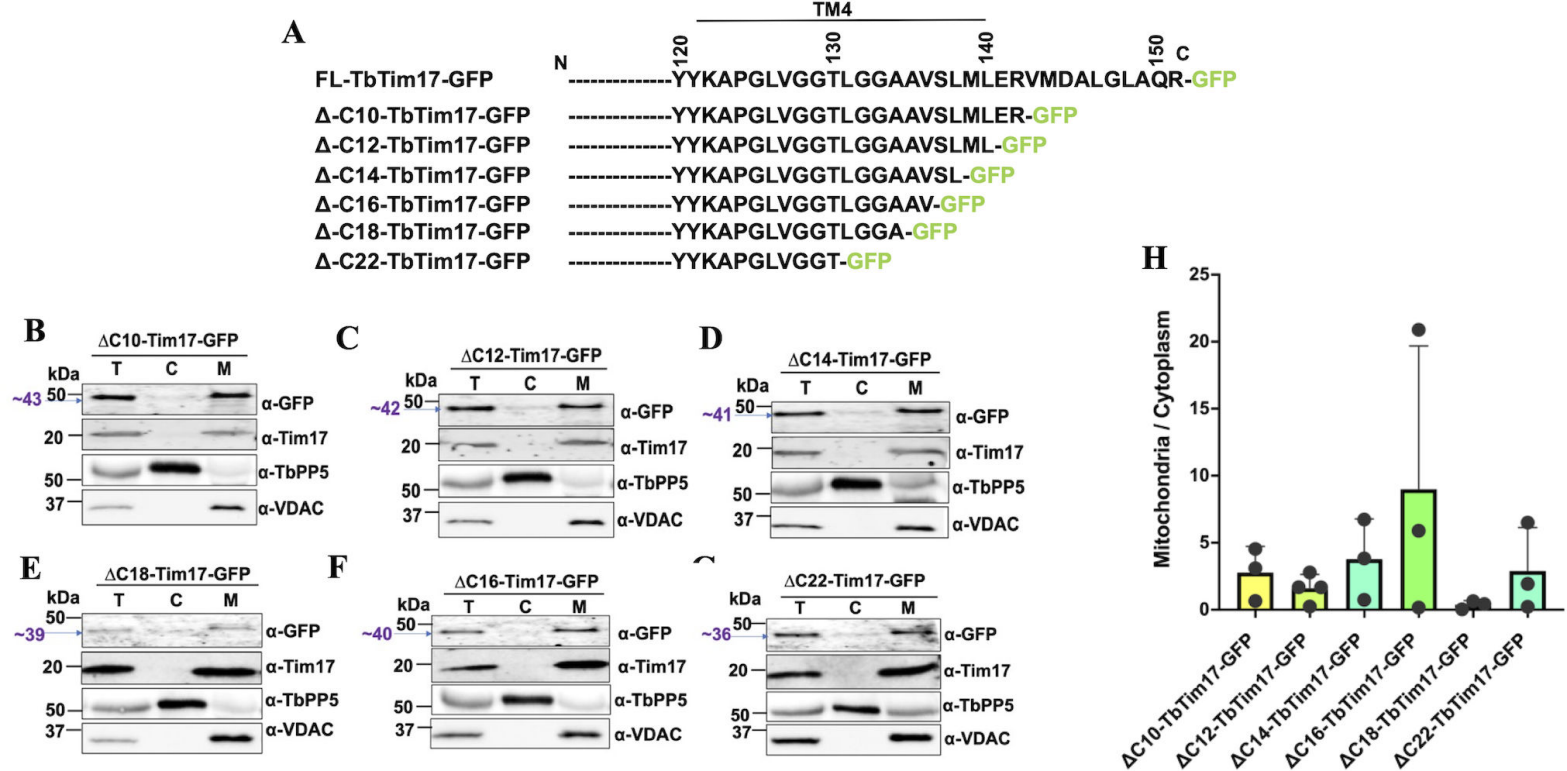
Sub-cellular location of the C-terminal deletion mutants of TbTim17. (**A**) Schematics of the C-terminal deletion mutants. (**B–G**) Immunoblot analysis of the sub-cellular fractions of *T. brucei*-expressed C-terminal deletion mutants of TbTim17. *T. brucei* cells expressing ∆C10-TbTim17-GFP (**B**), ∆C12-TbTim17-GFP (**C**), ∆C14-TbTim17-GFP (**D**), ∆C16-TbTim17-GFP (**E**), ∆C18-TbTim17-GFP (**F**), and ∆C22- TbTim17-GFP (**G**) were induced for 18–20 h, and sub-cellular fractions were collected. We found similar results for a longer induction period (40–45 h). Proteins in the total (T), cytosolic (C), and mitochondrial (M) fractions were analyzed by immunoblot using antibodies for GFP, TbTim17, TbPP5, and VDAC. (**H**) Densitometric analysis of the immunoblot results. Intensity of the GFP-fusion protein bands in the mitochondrial and cytosolic fractions was measured by Image Lab software (Bio Rad). The ratio of the mitochondrial vs cytosolic fractions was calculated and plotted against different cell types using GraphPad Prism. Error bars represent SEM for each data group. Sample size: *n* = 3 (in average).

**Fig 7:**
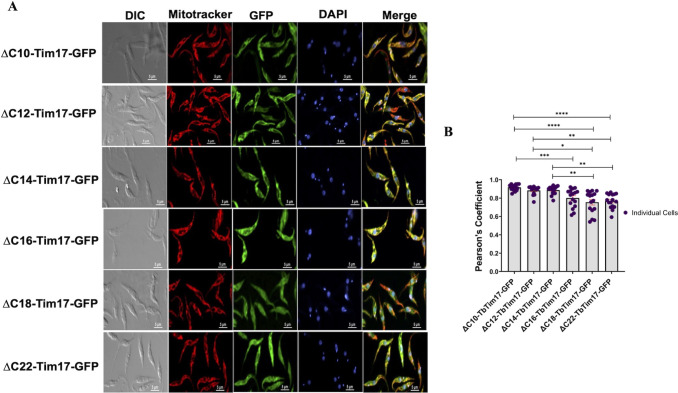
Immunofluorescence microscopy images of *T. brucei* expressing the C-terminal deletion mutants fused with GFP. (**A**) *T. brucei* cell lines, ∆C10-TbTim17-GFP, ∆C12-TbTim17-GFP, ∆C14-TbTim17-GFP, ∆C16-TbTim17-GFP, ∆C18-TbTim17-GFP, and ∆C22- TbTim17-GFP, were induced with doxycycline for 18–20 h. Cells were harvested and stained with MitoTracker Red to visualize the mitochondrion. Expression of GFP fusion proteins was seen by green fluorescence, DAPI was used to stain the nucleus and kinetoplast (mitochondrial genome), and phase-contrast pictures (DIC) are shown. Merge images show colocalization. (**B**) Pearson’s coefficient values were calculated from the merge images and plotted for each type of cells using GraphPad Prism. Individual data points (*n* = 10–20) are shown. Error bars represent SEM for each data group. Statistical significance was calculated by Kruskal-Wallis test; * indicates *P* = 0.0141; ** indicates *P* = 0.0086 (∆C12 vs ∆C22); *P* = 0.0036 (∆C14 vs ∆C18); *P* = 0.0020 (∆C14 vs ∆C22); *** indicates *P* = 0.001; **** indicates *P* < 0.0001.

Since we observed the C-terminal 31 amino acid residues are required for localization of TbTim17 into the mitochondria (Fig. 1 and 2), we wanted to investigate this region further to delineate the actual sequence responsible for mitochondrial targeting. For this purpose, we generated additional mutants by deleting two amino acid residues at a time starting from the 10 AAs of the C-terminal end, i.e., ΔC10-TbTim17-GFP (1–142 AAs), ΔC12-TbTim17-GFP (1–140 AAs), ΔC14-TbTim17-GFP (1–138 AAs), ΔC16-TbTim7-GFP (1–136 AAs), ΔC18-TbTim17-GFP (1–134 AAs), and ΔC22-TbTim17-GFP (1–130 AAs), and analyzed the localization pattern of these proteins by immunoblot analysis and confocal microscopy. Schematics of the mutants are shown in Fig. 6A. Our immunoblot results of the subcellular fractions revealed that all of these mutants were targeted to mitochondria, at comparable levels (Fig. 6B through G). The endogenous TbTim17 and VDAC were localized in the mitochondrial fraction, and TbPP5 was in the cytosolic fraction, as expected (Fig. 6B through G). Quantitation of the mitochondrial/cytosolic localization of the mutant proteins showed mitochondrial enrichment of the mutant proteins within two- to eightfold (Fig. 6H). The expression levels of ΔC18-TbTim17-GFP were very poor, suggesting the protein was unstable. We also performed immunofluorescence localization of these proteins by confocal microscopy and quantitated the PC values for colocalization (Fig. 7A and B). We found that ΔC10-TbTim17-GFP, ΔC12-TbTim17-GFP, and ΔC14-TbTim17-GFP mutants were colocalized with Mitotracker-stained mitochondria in most of the cells (Fig. 7A). The average PC values for the colocalization of ΔC10-TbTim17-GFP, ΔC12-TbTim17-GFP, and ΔC14-TbTim17-GFP were about 0.9 (Fig. 7B). However, ΔC16-TbTim17-GFP, ΔC18-TbTim17-GFP, and ΔC22-TbTim17-GFP colocalized with the Mitotracker-stained mitochondria in some cells and not in others (Fig. 7A). The average PC values for ΔC16-TbTim17-GFP, ΔC18-TbTim17-GFP, and ΔC22-TbTim17-GFP were 0.8, 0.78, and 0.8, respectively (Fig. 7B). Overall, we found that up to a 14 AAs deletion from the C-terminus had no effect; however, more than 14 AAs deletion had a slightly negative effect on TbTim17 localization in mitochondria. We showed that complete deletion of TM4 (deletion of 31 AAs from the C-terminus) disrupts mitochondrial localization of TbTim17 (Fig. 1 and 2). Therefore, these data together indicate that residues 120–132 AAs of TbTim17 likely have the critical information for mitochondrial targeting.

**Fig 8:**
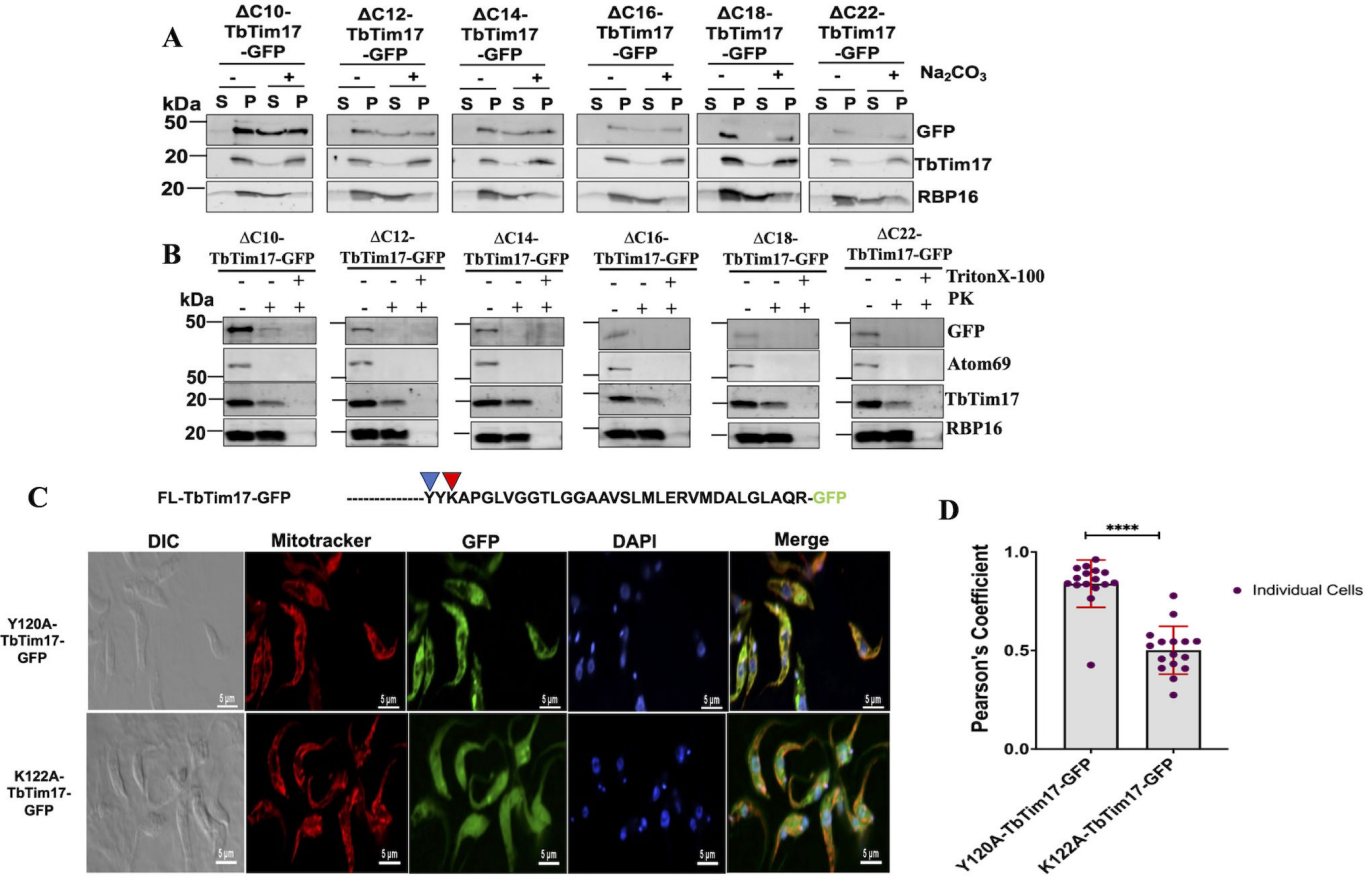
Sub-mitochondrial location of C-terminal deletion mutants and the effect of point mutations on mitochondrial localization of TbTim17. (**A**) Alkali extraction of mitochondria isolated from *T. brucei*-expressed ∆C10-TbTim17-GFP, ∆C12-TbTim17-GFP, ∆C14-TbTim17-GFP, ∆C16-TbTim17-GFP, ∆C18-TbTim17-GFP, and ∆C22- TbTim17-GFP. Mitochondria were treated (+) and left untreated (−) with Na_2_CO_3_, pH 11.0. The soluble (S) and insoluble pellet (P) fractions were separated by centrifugation and analyzed by immunoblot analysis using antibodies as indicated. (**B**) Limited protease digestion of mitochondria. Mitochondria from ∆C10-TbTim17-GFP, ∆C12-TbTim17-GFP, ∆C14-TbTim17-GFP, ∆C16-TbTim17-GFP, ∆C18-TbTim17-GFP, and ∆C22- TbTim17-GFP expressed *T. brucei* were treated (+) or left untreated (−) with proteinase K and TritonX-100 as shown. After treatment, mitochondria were recovered by centrifugation, and proteins were analyzed by immunoblot using antibodies as indicated. Each experiment was repeated three times, and representative blots are shown. (**C**) Sequence of the C-terminal region of TbTim17 and immunofluorescence microscopy images of *T. brucei*-expressed Y120A and K122A TbTim17-GFP. Induced cells were harvested and stained with MitoTracker Red to visualize the mitochondrion. Expression of GFP fusion proteins was seen by green fluorescence, DAPI was used to stain the nucleus and kinetoplast (mitochondrial genome), and phase-contrast pictures (DIC) are shown. Merge images show colocalization. (**D**) Pearson’s coefficient values were calculated from the merge images and plotted for each type of cells using GraphPad Prism. Individual data points (*n* = 10–20) are shown. Error bars represent SEM for each data group. Statistical significance was calculated by the Wilcoxon test; **** indicates *P* < 0.001.

We also wanted to see if these C-terminal deletion mutants were membrane integrated and entered into the mitochondria. Alkali extraction of the mitochondrial fractions showed that the ΔC10 to ΔC16 fusion proteins were partly present in the pellet and partly in the supernatant (Fig. 8A), indicating that the C-terminal end of the TM4 is needed for membrane integration of TbTim17. Interestingly, ΔC18-TbTim17-GFP and ΔC22-TbTim17-GFP were found mostly in the pellet fractions after alkali extraction (Fig. 8A), suggesting that deletion of the six AAs region (^131^LGGAAV^136^) relived the inhibition for membrane integration. PK protection assay showed that none of these C-terminal deletion mutants were protected from protease digestion, except ΔC10-TbTim17-GFP was partially protected (Fig. 8B), indicating that these mutant proteins did not cross the MOM, possibly stuck within the ATOM channel or attached to the MOM. Therefore, it showed that TM4 of TbTim17 is required for the entry into the mitochondria. In contrast, deletion of the 10 AAs hydrophilic region of the C-terminal minimally affected the import and integration of TbTim17 into the mitochondrial membrane. Together, these data show that the C-terminal end of the TM4 (^130^TLGGAAVSLMLER^142^) is not required for targeting but essential for translocation of TbTim17 through the MOM.

### Point mutations of Y^120^A did not, but K^122^A inhibits import of TbTim17 into mitochondria in *T. brucei*

From our observation, we found that 120–132 AAs of TbTim17 (Fig. 8C) possess information that are important for targeting and import of TbTim17 to mitochondria. Therefore, to identify critical AAs within this region, we performed site-directed mutagenesis analysis. Within this stretch of sequence, ^120^YYKAPGLVGGTLG^132^, there are very few polar residues, such as ^120^Y, ^121^Y, and ^122^K, the rest of the AAs are hydrophobic. We mutated two of these polar residues, ^120^Y and ^122^K, to A individually. Mutant clones were sequenced for further verification (Fig. S3A and B) and transfected to *T. brucei* to generate stable cell lines. Subcellular location of Y^120^A-TbTim17-GFP and K^122^A-TbTim17-GFP mutants was monitored by confocal microscopy. Results revealed that Y^120^A-TbTim17-GFP colocalized well with Mitotracker-stained mitochondria; however, K^122^A-TbTim17-GFP did not show good colocalization (Fig. 8D). The average PCs were observed 0.95 and 0.55, for Y^120^A-TbTim17-GFP and K^122^A-TbTim17-GFP, respectively (Fig. 8E). We also mutated G25A and G28A but did not see any effect on mitochondrial localization of TbTim17-GFP. Fig. 8E Overall, our results showed that the N-terminal part of the TM4 of TbTim17 and the single-charged residue K^122^ in loop 3 serve as an ITS and are critical for localization of TbTim17 to mitochondria.

**Fig 9:**
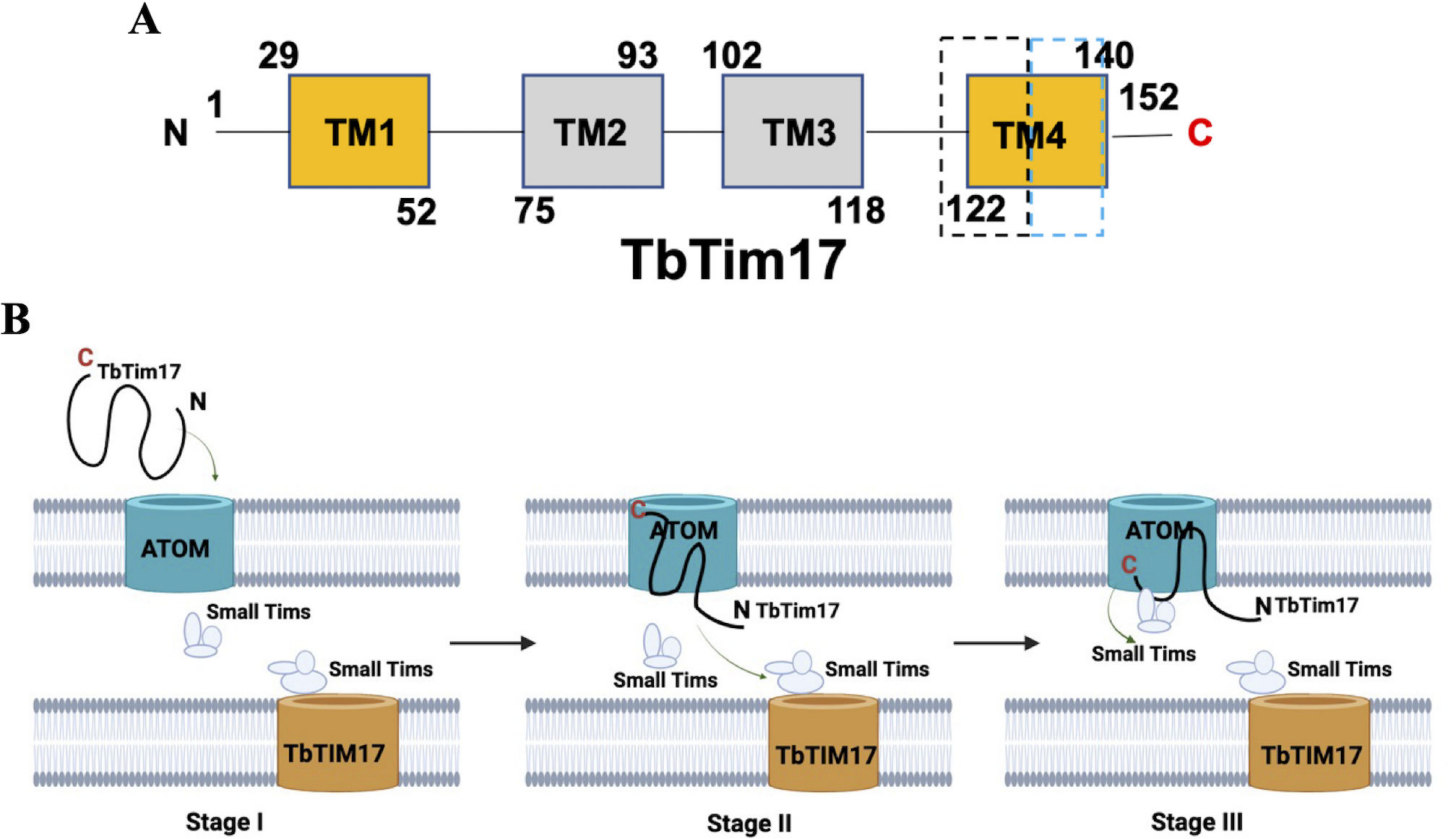
Schematics for the important structural domains of TbTim17 and its import process into the mitochondria. (**A**) A schematic for TbTim17 protein. Transmembrane domains (TM1–TM4) are in boxes. TM1 and TM4 (gold color) possess targeting information. The TM4 and a part of the linker region between TM3 and TM4 are divided into two boxes with dashed lines. The box with blue dashed line indicates the region that is not important for targeting but critical for TbTim17 translocation, whereas the box with purple dashed line indicates the region containing the ITS. (**B**) A working model of TbTim17 imports into the mitochondria. Stage I: ITSs located within TM1 and the region containing part of the TM4 + a part of the linker region (box with purple dashed line in panel A) are likely recognized by the ATOM receptors. Stage II: the N-terminal region translocates through ATOM, and the C-terminal region stays associated within the ATOM channel. Stage III: the part of the TM4 (box with blue dashed line in panel A) interacts with certain proteins, likely small TbTims, for further translocation from the ATOM.

Based on our results, we speculated a model for import of TbTim17 into mitochondria (Fig. 9). First, the ITSs located within TM1 and the region containing part of the TM4 + a part of the linker region (Fig. 9A) are likely recognized by the ATOM receptors. Next, the N-terminal region translocates through ATOM, and the C-terminal region stays associated within the ATOM channel (Fig. 9B). Finally, part of TM4 interacts with certain Tim subunits, likely small TbTims, for further translocation of TbTim17 from the ATOM (Fig. 9B).

## DISCUSSION

We have characterized at least two ITSs for TbTim17. We showed that the ITSs are within the (i) TM1 and in the (ii) C-terminal region that includes the third loop and TM4. Hydrophilic regions at the N- and C-termini, (1–30 AAs) and (140–152 AAs), respectively, are not required either for targeting or import of TbTim17 into the mitochondria. Both ITSs are required in combination for proper import and integration of TbTim17 into the MIM. Finally, K122, the single-charged residue within the third loop is critical for TbTim17 import. This is the first characterization of ITSs of a polytopic MIM protein in *T. brucei*.

The nuclear-encoded polytopic MIM proteins, such as mitochondrial carrier family proteins and the mitochondrial translocase proteins, Tim17/Tim22/Tim23 family, use ITSs for their import into mitochondria (49–53). A mostly studied protein in this regard is the mitochondrial ADP/ATP carrier in *S. cerevisiae*, known as AAC/ANT (54–56). The AAC protein has three modular domains, each consists of a pair of consecutive TMs and a linker region facing the matrix (51, 55, 57). Studies indicated that each module has independent targeting information, but they work in cooperation to translocate AAC through the TOM complex to the TIM22 complex (19, 55). Among these, the third module acts as the dominating factor for translocation of AAC (51). On the other hand, *in vitro* studies using isolated mitochondria and radiolabeled ScTim23 protein, Davis et al. showed that among four TMs, TM1 and TM4 of ScTim23 likely possess the targeting signals (44). However, TM1 and TM4 alone were poorly imported, not protected from protease digestion, and were not inserted into the MIM (44). The same study also showed that the positively charged residues within the connecting loops between two consecutive TMs (TM1 and TM2, and TM3 and TM4) are required for the insertion of Tim23 into the MIM but not for targeting to mitochondria (44).

Here, we investigated the targeting signal of TbTim17 using *in vivo* studies. First, we found that both TM1 and TM4 of TbTim17 have mitochondrial targeting information, and these signals individually were capable of translocating a reporter protein through the ATOM channel to a protease-protected location in mitochondria. However, neither of these TM-containing fusion proteins were inserted into the MIM, thus remaining as soluble proteins in the mitochondria. Second, our studies showed that TM4 along with TM3 and the linker between them (ΔN100-TbTim17-GFP) were not targeted to mitochondria. This indicates that the presence of TM3 may hinder the recognition of the signal in TM4 of TbTim17. Similarly, we found that the TM1-GFP [(30–50)-TbTim17-GFP] was imported, but addition of the N-terminal hydrophilic region to TM1 reduced targeting of the fusion protein [(1–50)-TbTim17-GFP]. This is in contrast to ScTim23, where TM1-TM2 and TM3-TM4 constructs were imported into mitochondria (44). Therefore, our results suggest that TbTim17 is likely imported not as loop structures as has been found for polytopic proteins in yeast. We postulated that for import and insertion of the full-length TbTim17 into the MIM, signals in TM1 and TM4 work in a cooperative manner possibly via sequential interaction with the translocase subunits. Third, we found that the region (130–152 AAs) of TbTim17 is dispensible for targeting. However, 130–142 AAs of TbTim17 are essential for its translocation through the MOM. It is likely that this region is needed to release the importing TbTim17 from the ATOM channel. Therefore, absence of this region caused the mutant proteins (ΔC12- to ΔC22-TbTim17-GFP) to be accessible to protease digestion. Thus, TM4 plays a dominant role for TbTim17 to cross the ATOM channel. Further deletion of the C-terminal region hampered the targeting and import of the mutant protein (ΔC31-TbTim17-GFP); thus, the protein was found in the cytosol. Therefore, the second ITS must be within 120–136 AAs of TbTim17. Within this region, we found that the single-charged residue, K122, is necessary for import of TbTim17 into the *T. brucei* mitochondrion. In contrast of having a single-charged residue in loop 3 of TbTim17, the equivalent region in ScTim17 and ScTim23 has multiple positively charged residues, and these were shown to be required for insertion of these proteins into the MIM (44). Therefore, the single-charged residue in TbTim17 performs a similar job, which could possibly be due to different architectures of the translocase complexes in this parasite. Together, we found that although there are some conserved features of ITSs in TbTim17 with that for ScAAC and ScTim23, the import process of TbTim17 appears distinct in *T. brucei*.

*T. brucei* ATOM has two receptor subunits, Atom46 and Atom69 (58). In our previous finding, we showed that TbTim17 interacts with both of these proteins (36, 59), suggesting that ITSs could be recognized either by Atom46 or Atom69, or by both at the same time. Recently, we also found that each of the small TbTims (TbTim9, TbTim10, TbTim11, TbTim12, TbTim13, and TbTim8/13) is capable of directly interacting with both the N- and C-terminal fragments of TbTim17, although at a greater extent with the later (60). In addition, we showed evidence that mitochondrial chaperone TbmHsp90/TbTRAP1 interacts with TbTim17 and is required for the assembly of the TbTIM17 complex (59). Altogether, we speculate that once the ITSs of TbTim17 are recognized by the Atom receptors, the N-terminus translocates through the ATOM channel, whereas the C-terminus stays attached to the Atom subunits. Subsequently, the C-terminus is released from the ATOM channel, and TbTim17 is translocated to the MIM with aid of small TbTims and by TRAP1. *T. brucei* only possesses a single TIM complex, TbTIM17. Therefore, it is likely that TbTim17 is finally translocated through this complex and assembled with other TbTim subunits via chaperons and assembly factors.

Overall, we identified the regions of TbTim17 that are crucial for TbTim17 import into mitochondria. Further investigation will identify the *T. brucei* proteins interacting with these regions and will reveal the import mechanism of an essential mitochondrial protein in *T. brucei*.

## MATERIALS AND METHODS

### Reagents

Hygromycin, geneticin (G418), phleomycin, and blasticidine were purchased from Invivogen; minimal essential medium and restriction enzymes were from Thermofisher; Mitotracker Red is from molecular probe; amino acids, doxycycline, and buffers were from Sigma-Aldrich.

### *T. brucei* strain and culturing

The procyclic form of the *T. brucei* 427 double resistant cell line (29-13) expressing a tetracycline repressor gene and a T7 RNA polymerase was grown in SDM-79 medium supplemented with 10% fetal bovine serum, G418 (15 µg/mL), and hygromycin (50 µg/mL) (61). Cells were inoculated at 2–3 × 10^6^/mL and allowed to grow in a tissue-culture flasks at 27°C incubator. Cell growth was monitored by counting cell number in a Neubauer hemocytometer under microscope.

### Plasmid constructs and transfection

To generate different deletion constructs for TbTim17, the corresponding regions of the TbTim17 open reading frame were PCR amplified with sequence-specific primers (Table S1). The forward and reverse primers were designed to add restriction sites for HindIII and XbaI at the 5′ ends. The PCR products were cloned in pRPX-GFP plasmid (62) within the HindIII and XbaI restriction sites. Plasmid DNA was linearized by NotI digestion and transfected into *T. brucei* 29-13 cells, as previously described (36). Transfected cells were selected with blasticidine (10 µg/mL).

### Protein structure modeling

Three-dimensional structure prediction tools, Raptor X server (63–68), was used to obtain predicted structures of the FL and truncated TbTim17 mutants. Structure homology modeling of the FL and TbTim17 mutants was performed using Swiss Model prediction software within Chimera X based on the cryo EM structure of HsTim22.

### Sub-cellular fractionation

Fractionation of *T. brucei* procyclic form cells was performed as described (69). Briefly, 2 × 10^8^ cells were pelleted and re-suspended in 500 µL of SMEP buffer (250 mM sucrose, 20 mM MOPS/KOH, pH 7.4, 2 mM EDTA, 1 mM phenyl methyl sulfonyl fluoride [PMSF]) containing 0.03% digitonin and incubated on ice for 5 min. The cell suspension was then centrifuged for 5 min at 6,800 × *g* at 4°C. The resultant pellet was considered as the crude mitochondrial fraction, and the supernatant contained soluble cytosolic proteins.

### SDS-PAGE and immunoblot analysis

Proteins from whole-cell lysates, cytosolic, or mitochondrial extracts were separated on a 12% Tris-SDS polyacrylamide gel, transferred to nitrocellulose membrane, and immunodecorated with polyclonal antibodies for TbTim17 (Tb927.11.13920) (41), VDAC (Tb927.2.2510) (70), TbPP5 (Tb927.10.13670) (71), RBP16 (Tb927.11.7900) (72), and mtHsp70 (Tb927.6.3740) (73). Antibodies for the GFP were purchased from ThermoFisher. Primary antibodies were diluted 1 in 1,000 in Tris-buffer saline containing 5% Tween 20. Blots were developed with anti-rabbit IgG secondary antibody conjugated with horse-radish peroxidase (Sigma) at 1 in 10,000 dilution, and the protein bands were developed with an enhanced Chemiluminescence Kit (Pierce).

### Alkali extraction

For sodium carbonate extraction, mitochondria (100 µg) were incubated with 100 µL of 100 mM sodium carbonate (pH 11.5) on ice for 30 min. The lysate was centrifuged at 14,000 × *g,* and the supernatant and pellet fractions were collected for further analysis.

### Proteinase K digestion

For limited PK digestion, mitochondria in SME buffer at 1 mg/mL concentration were treated with various concentrations of PK (0–200 μg/mL) for 30 min on ice. After incubation, PK was inhibited by PMSF (2 mM), and mitochondria were reisolated by centrifugation at 10,000 × *g* at 4°C for 10 min.

### Confocal microscopy

Live *T. brucei* cells (5 × 10^6^) expressing TbTim17 mutants were used for MitoTracker staining as previously described (69). Briefly, cells were washed twice with PBS and spread evenly over gelatin-coated slides. Once the cells had settled, the slides were washed with cold PBS to remove any unattached cells. The attached cells were fixed with 3.7% paraformaldehyde and permeabilized with 0.1% Triton X-100. After blocking with 5% non-fat milk for 30 min, the slides were washed with 1× PBS. DNA was stained with 1 µg/mL 4′,6-diamidino-2-phenylindole. Cells were imaged using a Nikon TE2000E widefield microscope equipped with a 60 × 1.4 NA Plan Apo VC oil immersion objective. Images were captured using a CoolSNAP HQ2 cooled CCD camera and Nikon Elements Advanced Research Software. There were no max projections of a *Z*-stack. The software used to calculate the Pearson’s coefficient is Nikon NIS Elements Advanced Research Imaging Software.

## Data Availability

All data are found within the paper and supplemental material.

## SUPPLEMENTAL MATERIAL

Supplemental Material msphere.00558-23-s0001.pdf Table S1; Figures S1-S4.

ASM does not own the copyrights to Supplemental Material that may be linked to, or accessed through, an article. The authors have granted ASM a non-exclusive, world-wide license to publish the Supplemental Material files. Please contact the corresponding author directly for reuse.
