# Supplementary material for: Distinct structural motifs are necessary for targeting and import of Tim17 in Trypanosoma brucei mitochondrion
Source: mSphere. 2024 Jan 9;9(1):e00558-23. doi: 10.1128/msphere.00558-23 (PMC10871166; doi:10.1128/msphere.00558-23)
Supplement: Supplemental Material — Table S1; Figures S1-S4. [file msphere.00558-23-s0001.pdf]

Supplementary materials

Distinct structural motifs are necessary for targeting and import of Tim17 in *Trypanosoma brucei* mitochondrion

Chauncey Darden[2], Joseph Donkor[1], Olga Korolkova[3], Muhammad Younas Khan Barozai[4], Minu Chaudhuri[1]*

Running Title: Internal Targeting Signals of TbTim17

[1]Department of Microbiology, Immunology, and Physiology, Meharry Medical College, Nashville, TN, 37208

[2]Department of Biochemistry, Cancer Biology, Neuroscience, and Pharmacology, Meharry Medical College, Nashville, TN, 37208

[3]The Consolidated Research Instrumentation, Informatics, Statistics, and Learning Integration Suite (CRISALIS), Meharry Medical College, Nashville TN, 37208

[4]Center for AIDS Health Disparites Research, Meharry Medical College, Nashville TN, 37208

* Corresponding Author: Minu Chaudhuri, PhD.

Phone: 615 327 5726

Email: mchaudhuri@mmc.edu

Table S1. Forward and Reverse Primers

Primer Name	Direction	Sequence* (5¢-3¢)
FL-TbTim17-GFP	Forward	5′-GATCAAGCTTATGACAACAATTCTCGACC-3′
FL-TbTim17-GFP	Reverse	5′-GATCTCTAGAGCGTTGAGCCAACCCCAATG-3′
ΔN50-TbTim17	Forward	5′-GATCAAGCTTATGTCAGCCGAATCCACAACG-3′
ΔN100-TbTim17	Forward	5′-GATCAAGCTTATGGATGTGTGGAATGCTAC-3′
ΔN120-TbTim17	Forward	5′-GATCAAGCTTATGTATAAAGCTCCCGGG-3′
ΔC10-TbTim17	Reverse	5′-GATCTCTAGAACGTTCAAGCATCAAAG-3′
ΔC12-TbTim17	Reverse	5′-GATCTCTAGAAAGCATCAAAGAAAC-3′
ΔC14-TbTim17	Reverse	5′-GATCTCTAGACAAAGAAACGGCTGC-3′
ΔC16-TbTim17	Reverse	5′-GATCTCTAGAAACGGCTGCACCACC-3′
ΔC18-TbTim17	Reverse	5′-GATCTCTAGATGCACCACCTAACG-3′
ΔC22-TbTim17	Reverse	5′-GATCTCTAGACGTACCACCCACAAG-3′
ΔC31-TbTim17	Reverse	5′-GATCTCTAGAATAACGACAGCCGTAC-3′
(1-30)-TbTim17	Reverse	5′-GATCTCTAGAAGCAGCTGGGGTAATG-3′
(30-50)-TbTim17	Forward	5′-GATCAAGCTTATGAACGTTGTTGGTG-3′
(30-50)-TbTim17	Reverse	5′-GATCTCTAGAAATCATTGCACCAAAGAGG-3′
$Y^{120}A$-TbTim17	Forward	5′-GCCCGGGAGCTTTATAAGCACGACAGCCGTA<br>CACTC-3′
$Y^{120}A$-TbTim17	Reverse	5′-GAGTGTACGGCTGTCGTGCTTATAAAGCTC<br>CCGGGC-3′
$K^{122}A$-TbTim17	Forward	5′-AGCCCGGGAGCTGCATAATAACGACAGCCGT<br>CACTC-3′
$K^{122}A$-TbTim17	Reverse	5′GAGTGTACGGCTGTCGTTATTATGCAGCTCCC<br>GGGCT-3′

*Restriction enzyme sites are underlined

Figure Legends

Figure S1. Structure models of the FL- and deletion mutants of TbTim17. (A) FASTA sequences for FL-TbTim17, ΔN50-TbTim17, ΔN100-TbTim17, ΔN120-TbTim17, and ΔC31-TbTim17 were subjected to analyze by the RaptorX software program. Output structures are shown. The N- and C-Terminals, transmembrane domains (TM1-TM4), and Loop regions (Loop1-Loop-3) are indicated. (B) Structural homology models for the TbTim17 FL and deletion mutants based on the cryo EM structure of HsTim22 were generated using Swiss Model prediction software within Chimera X. The N- and C-terminals are indicated by blue and red color, respectively.

Figure S2. Probability plot for sub-cellular localization of the C-terminal region of TbTim17 using TargetP 2.0 software program. The C-terminal region (120 – 152 AAs) was analyzed. The sequence of this region and the probability peaks are shown. The likelihood of being a signal peptide, mitochondrial targeting signal, or other as predicted for this region is shown in a table.

Figure S3. Probability plot for sub-cellular localization of the N-terminal fragments of TbTim17 using target 2.0 software program. The N-terminal regions (1-30 AAs), (30-50 AAs), and (1-50 AAs) were analyzed. The sequence of these regions and probability plots are shown. The likelihood of being a signal peptide, mitochondrial targeting signal, or others as predicted for each fragment is shown in tables.

Figure S4. Site-directed mutagenesis of Y120 and K122 to A in TbTim17. (A & B) The designed primers and sequences of the mutant clones for Y120A (A) and K122A (B) are shown. Positions of the primers and the mutant residues were highlighted by green and yellow, respectively.

Figure S1

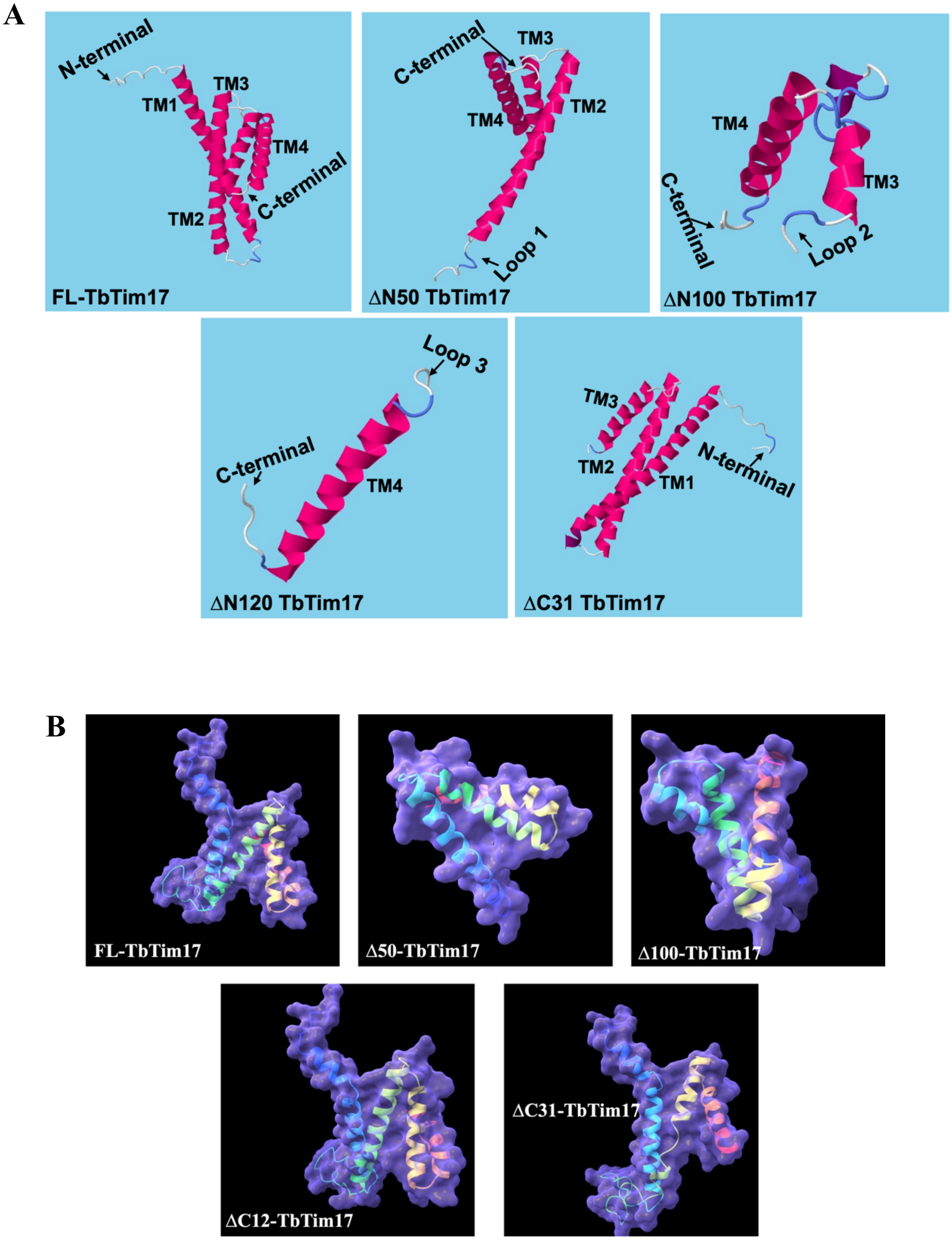
A
N-terminal
TM1
TM3
TM4
C-terminal
TM2
FL-TbTim17
C-terminal
TM3
TM4
TM2
Loop 1
ΔN50 TbTim17
TM4
TM3
C-terminal
Loop 2
ΔN100 TbTim17
Loop 3
C-terminal
TM4
ΔN120 TbTim17
TM3
TM2
TM1
N-terminal
ΔC31 TbTim17
B
FL-TbTim17
Δ50-TbTim17
Δ100-TbTim17
ΔC12-TbTim17
ΔC31-TbTim17

Figure S2

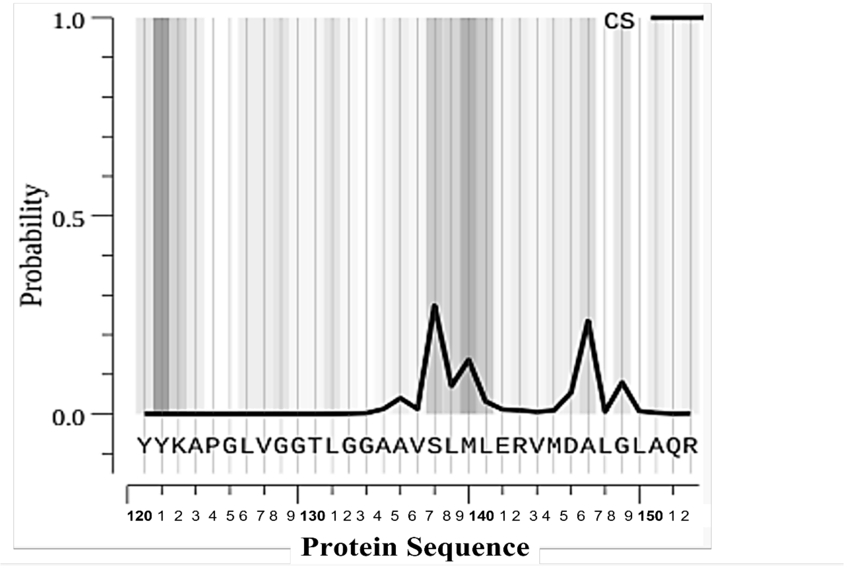

Sequence
Prediction: Signal peptide

CS pos: 18-19. AVS-LM. Pr: 0.2729

**Protein type**	**Other**	**Signal peptide**	**Mitochondrial transfer peptide**
**Likelihood**	0.1102	0.4843	0.4055

Figure S3

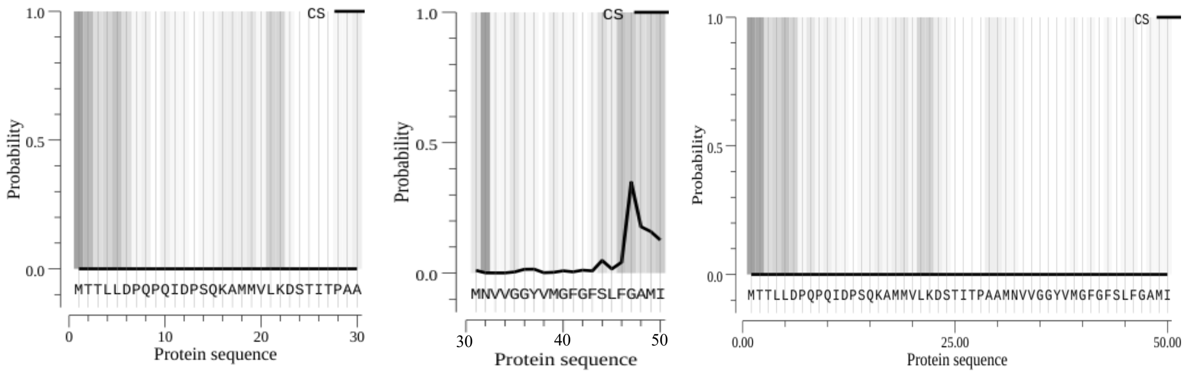

(1-30)-TbTim17 (30-50)-TbTim17 (1-50)-TbTim17

(1-30)-TbTim17

Sequence
Prediction: Other

Protein type	Other	Signal peptide	Mitochondrial transfer peptide
Likelihood	0.5743	0.4174	0.0083

(30-50)-TbTim17

Sequence
Prediction: Signal peptide

CS pos: 17-18. LFG-AM. Pr: 0.3476

Protein type	Other	Signal peptide	Mitochondrial transfer peptide
Likelihood	0.0842	0.8397	0.0761

(1-50)-TbTim17

Sequence
Prediction: Other

Protein type	Other	Signal peptide	Mitochondrial transfer peptide
Likelihood	0.8334	0.1523	0.0143

Figure S4

A

<table>
<tr><th>Primer Name Y120A-TbTim17</th><th>Primer-Template Duplex</th></tr>
<tr><td></td><td>gtggagtgtacggctgtcgttattataaagctcccgggcttg
  ||||||||||||||||| |||||||||||||||||
3'-ctcacatgccgacagcacgaatatttcgagggcccg-5'</td></tr>
</table>

>Tim17-Y120A-Forward_P1_B07.ab1
NNNNNNNNNNCANNTGNTCCCTCACAGAAGGCAATGATGGTGTTGAAGGACAGCACCATTACCCCAGCTGCTATGAACG
T TGTTGGTGGTTACGTAATGGGTTTTGGATTTTCCCTCTTTGGTGCAATGATTTCAGCCGAATCCACAACGCAGCGGATGG
GCACCGCCGACTTCTTCCGTTACAGTTTGAAAACGGCCCACAGACTCGGCTTCAGTTTTGCCTACTTTGGTTTCCTTTTC
GGTGGCATTGAGGTTGCGCTTGAGAAGCGACGGGGCCGTAAGGATGTGTGGAATGCTACACTTTCTGGTGGTCTTTTGGG
TGGAGTGTACGGCTGTCGTGCTTATAAAGCTCCCGGGCTTGTGGGTGGTACGTTAGGTGGTGCAGCCGTTTCTTTGATGC
TTGAACGTGTTATGGATGCATTGGGGTTGGCTCAACGCTCTAGAGTGAGCAAGGGCGAGGAGCTGTTCACCGGGGTGGTG
CCCATCCTGGTCGAGCTGGACGGCGACGTAAACGGCCACAAGTTCAGCGTGTCCGGCGAGGGCGAGGGCGATGCCACCTA
CGGCAAGCTGACCCTGAAGTTCATCTGCACCACCGGCAAGCTGCCCGTGCCCTGGCCCACCCTCGTGACCACCCTGACCT
ACGGCGTGCAGTGCTTCAGCCGCTACCCCGACCACATGAAGCAGCACGACTTCTTCAAGTCCGCCATGCCCGAAGGCTAC
GTCCAGGAGCGCACCATCTTCTTCAAGGACGACGGCAACTACAAGACCCGCGCCGANGTGAAGTTCGAGGGCGACACCCT
GGTGAACCGCATCGAGCTGAAGGGCATCGACTTCAAGGAGGACGGCAACATCCTGGGGCACAAGCTGGAGTACAACTACA
ACAGCCACAACGTCTATATCATGGCCGACAAGCAGAAGACGGCATCAAGGTGAACTTCAAGATCCGCCACAACATCGAGG
ACGGCAGCGTGCAGCTCGCCGACCACTACCAGCANACACCCCCAATCGGCGANGGCCCGNGCTGCTGCCCNANAACCAC
T ACCTGNANCACCCAGTCCGCCCTGA

B

<table>
<tr><th>Primer Name K122A-TbTim17</th><th>Primer-Template Duplex</th></tr>
<tr><td></td><td>gtggagtgtacggctgtcgttattataaagctcccgggcttgt
  ||||||||||||||||||||||| ||||||||||||
3'-ctcacatgccgacagcaataatacgtcgagggcccga-5'</td></tr>
</table>

Tim17-K122A-Forward_P1_C07.ab1
NNNNNNNNCNNNTGATCCCTCACAGAAGGCAATGATGGTGTTGAAGGACAGCACCATTACCCCAGCTGCTATGA
ACGTTGTTGGTGGTTACGTAATGGGTTTTGGATTTTCCCTCTTTGGTGCAATGATTTCAGCCGAATCCACAACGC
AGCGGATGGGCACCGCCGACTTCTTCCGTTACAGTTTGAAAACGGCCCACAGACTCGGCTTCAGTTTTGCCTAC
TTTGGTTTCCTTTTCGGTGGCATTGAGGTTGCGCTTGAGAAGCGACGGGGCCGTAAGGATGTGTGGAATGCTAC
ACTTTCTGGTGGTCTTTTGGGTGGAGTGTACGGCTGTCGTTATTATTGCAGCTCCCGGGCTTGTGGGTGGTACGT
TAGGTGGTGCAGCCGTTTCTTTGATGCTTGAACGTGTTATGGATGCATTGGGGTTGGCTCAACGCTCTAGAGTG
AGCAAGGGCGAGGAGCTGTTCACCGGGGTGGTGCCCATCCTGGTCGAGCTGGACGGCGACGTAAACGGCCAC
AAGTTCAGCGTGTCCGGCGAGGGCGAGGGCGATGCCACCTACGGCAAGCTGACCCTGAAGTTCATCTGCACCA
CCGGCAAGCTGCCCGTGCCCTGGCCCACCCTCGTGACCACCCTGACCTACGGCGTGCAGTGCTTCAGCCGCTA
CCCCGACCACATGAAGCAGCACGACTTCTTCAAGTCCGCCATGCCCGAAGGCTACGTCCAGGAGCGCACCATC
TTCTTCAAGGACGACGGCAACTACAAGACCCGCGCCGAGGTGAAGTTCGAGGGCGACACCCTGGTGAACCGCA
TCGAGCTGAAGGGCATCGACTTCAAGGAGGACGGCAACATCCTGGGGCACAAGCTGGAGTACAACTACAACAG
CCACAACGTCTATATCATGGCCGACAAGCAGAANAACGGCATCAAGGTGAACTTCANATCCGCCACAACATCGA
GGACGGCAGCGTGCAGCTCGCGACCACTACCAGCAGACACCCCATCGGCGACGGCCCGTGCTGCTGCCCGAN
NACCACTACCTGNAGCACCCAGTCCGCCCNGGANCAANNNNCCCNNCNNNNNAGCNNN
